# Loneliness and depression among older adults with cancer: an exploratory analysis of psychosocial constructs

**DOI:** 10.3389/fragi.2026.1821466

**Published:** 2026-07-23

**Authors:** Addison Kitrel, Ellen Park, Elizabeth Schofield, Caraline Demirjian, Nisha Mehta, Erica Fortune, Christian Nelson, Rebecca Saracino

**Affiliations:** 1 Department of Psychiatry and Behavioral Sciences, Memorial Sloan Kettering Cancer Center, New York, NY, United States; 2 Yeshiva University Ferkauf Graduate School of Psychology, New York, NY, United States; 3 Research and Training Institute, Cancer Support Community, Philadelphia, PA, United States; 4 Walther Center for Supportive Oncology, Indiana University Simon Comprehensive Cancer Center, Indianapolis, IN, United States

**Keywords:** anxiety, attitudes toward aging, demoralization, depression, geriatrics, loneliness, older adults with cancer, psychooncology

## Abstract

**Introduction:**

Loneliness is a common and clinically significant concern among older adults with cancer (OACs; age ≥70), who often experience heightened psychological distress compounded by aging-related challenges. The current study sought to identify the nature and magnitude of the relationships between loneliness and other psychosocial variables.

**Methods:**

Data were drawn from the baseline assessment of an ongoing randomized controlled trial of psychotherapy for distressed OACs. Loneliness was assessed using the PROMIS Social Isolation measure. Depressive symptoms were measured using the Hospital Anxiety and Depression Scale–Depression subscale and the Center for Epidemiologic Studies Depression Scale. Psychosocial factors included attitudes toward aging, demoralization, and anxiety. Fully adjusted regression models were conducted to examine the independent associations of loneliness and psychosocial factors with depressive symptoms while accounting for demographic covariates.

**Results:**

Among 258 participants, social isolation was positively associated with depressive symptoms at the bivariate level (CES-D: r =.694; p <.001; HADS-D: r =.629, p <.001). In fully adjusted models, social isolation (p <.001), anxiety (p = 0.015), and demoralization (p = 0.001) were independently associated with depressive symptoms.

**Discussion:**

Findings suggest that loneliness is independently associated with depressive symptoms among distressed OACs, even when accounting for other psychosocial variables and demographic characteristics. Anxiety and demoralization showed stronger and more consistent associations with depressive symptoms than attitudes toward aging. Although causal direction cannot be inferred from cross-sectional data, results highlight potential psychosocial targets for intervention and underscore the need for longitudinal research to clarify the dynamic relationships among loneliness, psychosocial factors, and depression in OACs.

## 1 Introduction

In the United States, there are an estimated 18.6 million cancer survivors, with predictions reaching above 26 million by the year 2040 (Wijnhoven et al., 2014; Tonorezos et al., 2024). Approximately two-thirds of all cancer survivors are 65 and older, and this number is expected to rise given the increasing population of older adults, advanced technology and earlier detection of cancer, and more effective, life-prolonging treatments (Kent et al., 2021). Although these developments reflect major progress in oncology practice and research, fewer funded studies that focus on survivorship care for older adults (Rowland et al., 2019), and even fewer have explored how aging-related stressors intersect with the psychosocial challenges of cancer survivorship (Leach et al., 2016).

Loneliness, defined as the subjective negative experience of inadequate or insufficient social connection, is an important psychosocial factor associated with adverse health outcomes (Hawkley and Cacioppo, 2010). In a recent 2023 report, the U.S. Surgeon General declared loneliness and social isolation as profound public health threats (U.S. Public Health Service, 2023), increasing the risk of all-cause mortality by 25% and 29%, respectively (Holt-Lunstad et al., 2015). An estimated 35.9% of adult cancer survivors report persistent loneliness and rates may be even higher among those coping with cancer due to treatment-related isolation, reduced mobility, changes in social support networks, and aging-related stressors (Wheldon et al., 2025). Moreover, loneliness in later life has been linked to poorer physical health, cognitive decline, functional impairment, and increased mortality risk, underscoring its significance as a public health issue (Lyu et al., 2024). It is a particularly salient concern within the context of geriatric oncology survivorship.

Depression is also common among individuals with cancer, with older adults experiencing unique risk factors related to multimorbidity, functional limitations, and existential concerns (Weinberger et al., 2011). In fact, depressive symptoms in older adult cancer survivors (OACs) are associated with diminished quality of life, poorer treatment adherence, increased healthcare utilization, and elevated mortality (Mausbach and Irwin, 2017). Although loneliness consistently remains the most robust predictor of depression across the lifespan (Lee et al., 2021), the psychosocial correlates of this construct and how it co-occurs with depression in OACs remain insufficiently understood.

Recent research suggests that several psychosocial processes may help explain the relationship between loneliness and depression. For example, negative attitudes toward aging, such as internalized ageism or loss or purpose, have been associated with poorer psychosocial outcomes and may amplify the psychological impact of loneliness (Ribeiro-Goncalves et al., 2023). Within the context of psychosocial oncology, demoralization, a state of hopelessness and loss of meaning, purpose, or courage, and anxiety have been independently associated with both loneliness and depression and may further compound emotional distress in OACs (Mehnert et al., 2011; Arvanitou et al., 2023; Pilleron et al., 2023). However, few studies have examined these psychosocial factors simultaneously within a single multivariable framework in OACs, limiting the ability to determine independent contributions when considered together.

To address this research gap, the present study examined associations among loneliness, depressive symptoms, and multiple psychosocial factors (e.g., anxiety, demoralization, and attitudes toward aging) within a single multivariable framework in a clinically distressed sample of OACs. Rather than testing specific causal pathways, this approach allows for the identification of psychosocial factors that are independently associated with depressive symptoms after accounting for demographic characteristics and interrelated psychosocial variables. In addition, this study extends the literature by using the PROMIS Social Isolation scale as validated measure of perceived social isolation, which and aligns closely with contemporary conceptualizations of loneliness.

The aims of this study are to: (1) examine associations between key demographic and clinical factors and psychosocial variables; (2) evaluate bivariate relationships among loneliness, depressive symptoms, and psychosocial factors; (3) determine whether loneliness is independently associated with depressive symptoms in multivariable models adjusting for demographic and psychosocial covariates; and (4) examine the relative magnitude of associations between psychosocial variables and loneliness and depressive symptoms within adjusted regression models.

## 2 Materials and methods

### 2.1 Study design

This study is a secondary analysis of baseline survey data from the Cancer and Aging: Reflections for Elders (CARE) study, a large randomized controlled trial recruiting participants across the United States. The CARE intervention is a 9-session geriatric-specific psychotherapy for older adults with cancer and is being compared with Social Work and Supportive Counseling (SWSC). The present analyses are based solely on baseline data and are independent of the trial’s intervention outcomes. This study has been approved by the Memorial Sloan Kettering Cancer Center’s (MSKCC) Institutional Review Board (20-191). All participants provided informed consent prior to participation in study procedures.

### 2.2 Study participants

Participants were screened for eligibility prior to enrollment by trained research staff. Screening included verification of inclusion and exclusion criteria and assessment of capacity to provide informed consent. Individuals who did not meet eligibility criteria or who were unable to provide informed consent were not enrolled in the study. Eligible participants were then invited to provide informed consent prior to any study procedures. Following consent, participants were enrolled in the parent trial and completed baseline assessments.

Eligible participants met the following inclusion criteria: 1) 70 years or older, 2) a diagnosis of cancer, 3) receiving active treatment, received active treatment in the past 6 months, or currently on active surveillance with follow-ups at least every 6 months, 4) fluent in English, 5) a Hospital Anxiety and Depression Scale (HADS) of 6 or greater on HADS-D or 6 or greater on HADS-A or a score of 4 or greater on the Distress Thermometer, 6) a score of 60 or greater on the Karnofsky Performance Rating Scale (KPRS), 7) a score of 11 or lower on the Blessed Orientation-Memory-Concentration Scale (BOMC), and 8) provided complete data on outcome variables. Participants were excluded from the study if they were: 1) currently taking antidepressant medication for less than 3 months, 2) currently receiving hospice care, 3) unable to provide informed consent and complete study sessions/assessment, or 4) had current untreated major psychotic disorder.

The sample size for the present study was determined by the recruitment and enrollment targets of the parent randomized controlled trial. As this manuscript represents a secondary analysis of baseline data, no separate *a priori* sample size calculation was conducted for the present analyses.

### 2.3 Data collection procedure

Participants were recruited using a consecutive sampling approach as part of an ongoing randomized controlled trial. Three main recruitment strategies were employed: 1) direct patient recruitment at MSKCC via letters, emails, and phone call follow-ups; 2) local Cancer Support Community (CSC) and Gilda’s Club (GC) outreach via flyers and emails, radio announcements, and newspaper ads; and 3) national recruitment through the CSC National Headquarters (CSC HQ) Nationwide Online Digital Community via social media posts, online newsletters, and email blasts. The Clinical Research Coordinator (CRC) contacted interested participants to verify eligibility and all eligible individuals who met inclusion criteria during the recruitment period were approached for participation.

### 2.4 Measures

Demographic and clinical characteristics. Participant characteristics including age at enrollment, race, ethnicity, gender, relationship status, educational attainment, disease type (i.e., solid tumor vs. non-tumor), and primary cancer site were collected via baseline surveys at the time of study enrollment. For the purposes of this analysis, race, relationship status, and education were dichotomized as follows: race (0 = White, 1 = non-White), relationship status (0 = unpartnered, 1 = married/partnered), and education (0 = college or more, 1 = less than college).

Loneliness. Loneliness was measured using the Patient-Reported Outcomes Measurement Information System (PROMIS) Social Isolation–Short Form 8a. This is an 8-item questionnaire that assesses the perception of the degree of isolation and perceptions of being avoided, excluded, detached, or disconnected from others on a scale from 1 (“Never”) to 5 (“Always”). This scale has good reliability (Cronbach’s alpha = 0.86) (Kittel et al., 2024) and has been used to assess loneliness in cancer survivors (Clifton et al., 2022).

Attitudes toward Aging. Attitudes toward aging were assessed using the Attitudes to Aging Questionnaire (AAQ), a 24-item measure consisting of a three-factor model to encompass psychological growth, psychosocial loss, and physical change (Laidlaw et al., 2007). For this study, we utilized the 8-item psychological growth subscale, focusing on themes such as wisdom, coping, acceptance, positive appraisal, and personal growth. Each item is scored on a 5-point Likert scale (1-5), yielding a range of 8–40.

Demoralization. The 24-item Demoralization Scale (DS) measures existential distress across five dimensions: loss of meaning and purpose, dysphoria, disheartenment helplessness, and a sense of failure (Kissane et al., 2004). Items are rated on a 5-point Likert scale ranging from 0 (“Never”) to 4 (“All the time”). A total score is obtained by summing up the single scale scores. The DS has been widely utilized in the psychosocial oncology literature and has good internal consistency and reliability (Kissane et al., 2004; Mehnert et al., 2011).

Anxiety. Anxiety was measured using the Patient-Reported Outcomes Measurement Information System (PROMIS) Anxiety Short Form 8a. The items assess fear, anxious misery, hyperarousal, and somatic symptoms related to arousal. Items are rated on a 5-point Likert scale ranging from 1 (“Not at all”) to 5 (“Very much”). The PROMIS-Anxiety scale has been widely used in cancer populations and has good internal consistency with a Cronbach’s alpha of greater than 0.89 (Recklitis et al., 2021; Crane et al., 2019).

Depressive symptoms. The Hospital Anxiety and Depression Scale (HADS) consists of one subscale to identify and quantify depression (HADS-D) in physically ill patients (Zigmond and Snaith, 1983). The HADS-D subscale consists of 7 items and is on a four-point Likert scale (range 0–3) and the score is the sum of the respective seven items, ranging from 0 to 21. This scale has good reliability (Cronbach’s alpha = 0.86 for depression) (Montazeri et al., 2003) and has been widely used to assess anxiety and depression in cancer survivors (Hartung et al., 2017). Depressive symptoms were also measured using the 20-item Center for Epidemiologic Studies Depression (CES-D) Scale, with each item scored on a four-point Likert scale from 0 (rarely/none of the time) to 3 (most/all of the time) with a range of 0–60 points (Hann et al., 1999). Participants rated the frequency with which they had been bothered by depressive symptoms over the past week. The CES-D has good internal consistency, with Cronbach’s alpha between 0.79 and 0.92 (Wijnhoven et al., 2014).

### 2.5 Statistical analysis

Descriptive statistics were computed to characterize the sample and loneliness, attitudes toward aging, demoralization, anxiety, and depressive symptom outcomes. The primary outcomes for the analysis were the depression measures: CES-D and HADS-D. Preliminary analyses were conducted to identify demographic covariates for inclusion in the multivariable models. Demographic variables (Table 1) were examined in relation to depressive symptoms using appropriate bivariate analyses. Variables significantly associated with at least one depressive symptom outcome (p < 0.05) were retained as covariates for modeling analyses (Table 2). Accordingly, age, gender, race, and relationship status were included as covariates in the fully adjusted models, whereas education and disease site were not retained due to a lack of significant bivariate relationship with either depressive outcome. Age was analyzed using Pearson correlation and dichotomized demographic and clinical variables using a series of independent t-tests. Analyses were conducted using pairwise deletion, such that all available data were used for each statistical test. Pearson correlations were computed to examine bivariate associations among psychosocial factors. Multiple linear regression analyses were then conducted to examine whether loneliness was uniquely associated with depressive symptoms after controlling for covariates and other psychosocial factors, with separate models estimated for each depression outcome. Standardized regression coefficients (β) were used to compare relative strength among predictors. Assumptions underlying Pearson correlations, independent samples t-tests, and multiple linear regression analyses were evaluated prior to hypothesis testing. These included assessment of normality and linearity for continuous variables, as well as homogeneity of variance for group comparisons. Multicollinearity was assessed using variance inflation factors (VIF), with VIF values ≥5 considered indicative of potential multicollinearity. All assumptions were determined to be acceptable for the analyses performed. All analyses were conducted using IBM SPSS Statistics (version 31).

**TABLE 1:** Patient characteristics (n = 258).

Variable	n (%)
Age, mean (SD)	74.6 (4.17)
Gender, % Female Male	198 (76.8) 60 (23.2)
Race, % White People of color (POC)	227 (88.0) 31 (12.0)
Ethnicity, % Hispanic Non-Hispanic	8 (3.10) 250 (96.9)
Relationship status, % Partnered Unpartnered	145 (56.0) 113 (44.0)
Educational attainment, % College or more Less than college	197 (76.4) 61 (23.6)
Disease site, % Solid tumor Non-solid tumor	191 (74.1) 67 (25.9)
Loneliness, Mean (SD)	12.1 (5.19)
Attitudes toward aging, Mean (SD)	30.9 (4.72)
Demoralization, Mean (SD)	35.3 (11.7)
Anxiety, Mean (SD)	18.7 (6.33)
Depressive symptoms, Mean (SD) CES-D HADS-D	18.8 (10.1) 6.71 (3.59)

**TABLE 2:** Correlations and group mean (SD) by demographic characteristic.

Characteristic	Loneliness	Attitudes toward aging	Demoralization	Anxiety	CES-D	HADS-D
Age, corr	−0.145*	0.100	−0.125*	−0.066*	−0.142*	−0.135*
Gender, Female Male	12.8 (5.19) *** 9.73 (4.48)	30.7 (4.80) 31.6 (4.41)	36.5 (11.4)** 31.4 (11.8)	19.3 (6.40)** 16.8 (5.72)	19.6 (10.0)* 16.0 (9.90)	7.06 (3.69)** 5.55 (2.98)
Race, White POC	12.1 (5.15) 12.1 (5.59)	30.7 (4.67)* 32.5 (4.87)	35.6 (11.6) 33.1 (12.5)	18.7 (6.07) 18.5 (8.09)	18.4 (9.62) 21.1 (12.9)	6.57 (3.51) 7.71 (4.05)
Ethnicity, Hispanic Non-Hispanic	11.3 (4.83) 12.1 (5.21)	33.5 (4.66) 30.8 (4.71)	34.0 (10.7) 35.4 (11.8)	19.6 (6.05) 13.7 (6.35)	17.9 (11.4) 18.8 (10.1)	5.13 (3.80) 6.76 (3.58)
Relationship status, Partnered Unpartnered	10.6 (4.79)*** 14.0 (5.08)	31.2 (4.40) 30.5 (5.10)	33.6 (10.8)** 37.6 (12.4)	18.5 (6.57) 19.0 (6.03)	17.6 (9.75)* 20.2 (10.3)	6.27 (3.55)* 7.26 (3.58)
Education, College or more Less than college	12.3 (5.10) 11.3 (5.47)	30.8 (4.74) 31.2 (4.72)	36.0 (11.5) 33.0 (12.1)	18.9 (6.17) 17.9 (6.82)	19.0 (9.94) 17.8 (10.6)	6.96 (3.57) 5.83 (3.57)
Disease site, Solid tumor Non-solid tumor	12.0 (5.28) 12.2 (4.96)	30.8 (4.87) 31.3 (4.30)	34.6 (11.3) 37.3 (12.5)	18.6 (6.37) 19.0 (6.23)	18.6 (9.93) 19.2 (10.6)	6.63 (3.64) 6.91 (3.46)

*p < 0.05, **p < 0.01, ***p < 0.001 indicate statistically significant differences between groups. For age, Pearson correlations are calculated for each outcome. For dichotomous outcomes, independent samples t-tests are used with means for each group reported.

## 3 Results

### 3.1 Demographics

Descriptive statistics for demographic and clinical characteristics are presented in Table 1. Participants had a median age of 74 years. The sample was predominantly female (76.8%), white (88.0%), and non-Hispanic (96.9%). Slightly over half of the participants were partnered (i.e., married or in a domestic partnership; 56.0%).

Descriptive statistics for primary study variables are presented in Table 1. The mean PROMIS Social Isolation raw score was 12.1 (SD = 5.19). The mean score on the CES-D was 18.8 (SD = 10.1) and 6.71 (SD = 3.59) on the HADS-D. Notably, the mean scores for both CES-D and HADS-D fell above the optimal cutoff scores (≥15 and ≥6 respectively) for detecting Major Depressive Disorder (MDD) in older adults with cancer, consistent with the distressed nature of the sample (Saracino et al., 2016). Demoralization scores were elevated relative to established clinical cutoffs, with a mean Demoralization Scale score of 35.3 (SD = 11.7) (Kissane et al., 2004). The mean PROMIS Anxiety raw score was 18.7 (SD = 6.33). Attitudes toward aging reflected generally positive perceptions (M = 30.9, SD = 4.72). Distributions of variables were approximately normal, with no substantial skewness observed.

### 3.2 Bivariate analyses

The results of the bivariate analyses for demographic and clinical characteristics are included in Table 2. Age was significantly associated with depressive symptoms, such that older age was associated with lower levels of depressive symptoms for both CEDS-D (*r* = −0.142, *p* = 0.022) and HADS-D (*r* = −0.135, *p* = 0.030). Gender, race (collapsed), and relationship status (collapsed) were significantly associated with at least one factor or outcome variable. Other categorical variables were not significantly associated with these variables.

Bivariate relationships among loneliness, depressive symptoms, and various psychosocial mediators are given in Table 3. Loneliness was positively correlated with CES-D (*r* = 0.694, *p* < 0.001) and HADS-D (*r* = 0.629, *p* < 0.001) scores, as well as with demoralization (*r* = 0.745, *p* < 0.001) and anxiety (*r* = 0.492, *p* < 0.001). Loneliness was negatively correlated with attitudes toward aging (*r* = −0.362, *p* < 0.001), such that loneliness was associated with more negative attitudes towards aging. Each psychosocial factor was significantly associated with depressive symptoms on both the HADS-D and CES-D, with demoralization and anxiety demonstrating the strongest associations with CES-D scores.

**TABLE 3:** Bivariate correlations among study variables.

Study variable and measure	1	2	3	4	5	6
1. Loneliness: PROMIS social isolation short form 8a	-	−0.36^***^	0.76^***^	0.49^***^	0.69^***^	0.63^***^
2. Attitudes towards aging: Attitudes to aging (psychological growth subscale)	​	-	−0.41^***^	−0.25^***^	−0.40^***^	−0.36^***^
3. Demoralization: Demoralization scale	​	​	-	0.59^***^	0.73^***^	0.63^***^
4. Anxiety: PROMIS anxiety short form 8a	​	​	​	-	0.66^***^	0.48^***^
5. Depression: CES-D score	​	​	​	​	-	0.78^***^
6. Depression: HADS-D score	​	​	​	​	​	-

*** indicates p < 0.001 (2-tailed).

### 3.3 Regression analyses

Loneliness was uniquely associated with depressive symptoms as measured by CES-D (β = 0.592, *SE* = 0.075, *t* = 10.223, *p* < 0.001) and HADS-D (β = 0.649, *SE* = 0.037, *t* = 12.138, *p* < 0.001). Loneliness accounted for an additional ΔR^2^ = 0.293 of variance in CES-D scores and ΔR^2^ = 0.352 of variance in HADS-D scores beyond the covariates.

For depressive symptoms as measured by CES-D, the fully adjusted model accounted for 66.2% of the variance in depressive symptoms. Loneliness was independently associated with depressive symptoms (β = 0.292, p < 0.001), as was anxiety (β = 0.333, p < 0.001), demoralization (β = 0.286, p < 0.001), and attitudes toward aging (β = −0.087, p = 0.032), controlling for age, gender, and relationship status (Table 4).

**TABLE 4:** Adjusted models of depressive symptom outcomes.

Variable	CES-D (n = 258)			HADS-D (n = 258)		
	B (SE)	β	p	B (SE)	β	p
Loneliness	0.564 (0.114)	0.292	<0.001	0.236 (0.051)	0.342	<0.001
Attitudes to aging	−0.186 (0.086)	−0.087	0.032	−0.069 (0.039)	−0.090	0.076
Demoralization	0.246 (0.052)	0.286	<0.001	0.075 (0.023)	0.245	0.001
Anxiety	0.529 (0.073)	0.333	<0.001	0.080 (0.032)	0.141	0.015

The model adjusted for age, gender, race, and relationship status.

In the fully adjusted model predicting HADS-D scores, the overall model was significant, *F* (7, 250) = 31.66, *p* < 0.001, accounting for 47.0% of the variance in depressive symptoms (adjusted *R* ^2^ = 0.455). Loneliness was independently associated with HADS-D scores (β = 0.342, *p* < 0.001). Anxiety (β = 0.141, *p* = 0.015) and demoralization (β = 0.245, *p* = 0.001) also demonstrated independent associations with depressive symptoms, controlling for age, gender, and relationship status. Attitudes toward aging (β = −0.090, *p* = 0.076) was not independently associated with depressive symptoms upon inclusion of the other variables (Table 4).

## 4 Discussion

The present study examined associations among loneliness, other key psychosocial variables, and depressive symptoms in a large, distressed sample of older adults with cancer (OACs). Using both bivariate and fully adjusted multivariable analyses, findings indicate that loneliness is strongly associated with depressive symptoms and that this association persists even when accounting for demographic characteristics and key psychosocial factors. Anxiety and demoralization emerged as particularly salient correlates of depressive symptoms, whereas attitudes toward aging were less consistently associated once other variables were considered simultaneously. Overall, these findings underscore the complexity of emotional distress in OACs and support the development of multicomponent interventions that address loneliness alongside existential distress, anxiety, and maladaptive attitudes toward aging and may be relevant to depressive symptom burden in OACs.

Loneliness was positively associated with depressive symptoms at the bivariate level across both depression measures. This finding reinforces the conceptualization of loneliness as a central component of emotional distress in later life, particularly among populations facing cancer- and age-related challenges such as physical decline, role loss, treatment-related changes and other shifts in social support networks (Wheldon et al., 2025). Importantly, loneliness remained independently associated with depressive symptoms in fully adjusted models that included anxiety, demoralization, attitudes toward aging, and demographic covariates, suggesting that loneliness captures a distinct aspect of psychological distress not fully explained by related affective constructs. Future longitudinal research should elucidate the temporal relationships between loneliness and depression among OACs. The relationship is likely bidirectional, with loneliness and depressive symptoms potentially co-occurring and being mutually associated.

Anxiety and demoralization demonstrated strong and independent associations with depressive symptoms in fully adjusted models, highlighting their clinical relevance in this population. Anxiety is characterized by heightened uncertainty and fear, and is associated with greater depressive symptoms, particularly in the context of cancer-related uncertainty and aging. Among OACs, loneliness may be a marker for heightened anxiety related to health outcomes, amplify fears related to disease progression, functional decline, or dependency, increasing emotional distress and vulnerability to depression. Furthermore, anxiety may be associated with reduced engagement in social or health-promoting behaviors, and may co-occur with a cycle of increasedloneliness and low mood. As such, interventions that incorporate anxiety management strategies, such as cognitive-behavioral techniques, mindfulness-based stress reduction, or skills for tolerating uncertainty and enhancing coping, may help reduce depressive symptom burden in individuals reporting higher levels of loneliness (Tutino et al., 2022; Chen et al., 2025). In parallel, interventions that help patients increase opportunities for rewarding social interactions, such as Behavioral Activation (Saracino et al., 2024) could also be associated with lower anxiety and depressive symptoms among OACs.

Unlike depression alone, demoralization is characterized by a sense of hopelessness, helplessness, inability to cope due to a loss of meaning, sense of failure, and loss of purpose in life, which may be especially relevant in later life and during serious illness, such as cancer (Kaneriya et al., 2023). These findings suggest that interventions aimed at establishing or reestablishing meaning, agency, and hope may be effective for OACs who are socially isolated. Specifically, meaning-centered psychotherapy, dignity therapy, and interventions focused on identifying values may be associated with lower demoralization and depressive symptoms (Chochinov et al., 2005; Breitbart et al., 2012; Sethi et al., 2020).

While attitudes toward aging were associated with depressive symptoms at the bivariate level, they were not consistently associated in fully adjusted models. While attitudes toward aging were independently associated with CES-D scores, this association did not remain significant in the HADS-D model, suggesting the relationship with depression symptoms may be dependent on the measure used and symptom profile assessed. The CES-D assesses a broad range of depressive symptomatology, including affective distress, somatic complaints, and interpersonal difficulties, whereas the HADS-D was designed to minimize the influence of somatic symptoms and more specifically captures core features of depression such as anhedonia. It is therefore possible that negative attitudes toward aging are more strongly associated with the broader affective and somatic dimensions of depression captured by the CES-D, particularly in the context of cancer-related physical decline, fatigue, and functional limitations. More broadly, feelings of social disconnection may reinforce perceptions of aging as a period of decline, loss of independence, or diminished social value, which may contribute to emotional distress in a more global, rather than symptom-specific manner. Negative perceptions of aging may also be associated with depressive symptoms indirectly through broader emotional distress or existential vulnerability, rather than functioning as an independent correlate when anxiety and demoralization are considered simultaneously. Alternatively, attitudes toward aging may reflect a more nuanced pattern of association with psychological wellbeing that is not fully captured in cross-sectional models. Interventions that target maladaptive aging perceptions, such as cognitive-behavioral strategies, meaning-centered psychotherapies, or psychoeducation that challenges ageist beliefs and promotes adaptive narratives of aging, may still help buffer the depressive impact of loneliness (Haase et al., 2022; Sirois et al., 2025). These findings underscore the importance of examining multiple psychosocial factors concurrently to clarify their relative associations to depressive symptoms in older adults with cancer.

### 4.1 Limitations and future directions

There are a number of limitations to the present study. First, these findings are based on cross-sectional baseline data, and thus, suggestions of causation should be made with caution. Additionally, sample characteristics limit generalizability. Most participants were White, female and college-educated, and were all enrolled in a psychotherapy research trial that required endorsement of clinically significant distress, which may reflect a select subset of OACs who are more distressed, and more open to discussing emotional concerns or actively seeking mental health support. Thus, these findings may not be generalizable to more diverse racial or ethnic groups, to men, or to OACs who are less distressed or engaged in psychosocial support. It would be important to examine the cultural factors influencing social isolation, aging perceptions, and help-seeking behaviors as well.

Limitations notwithstanding, the present study provides further evidence that loneliness and depression are closely intertwined in OACs and associated with multiple psychosocial factors within a single multi-variable framework. By moving beyond bivariate associations and examining independent relationships in fully adjusted models, this study provides a more nuanced understanding of how loneliness and related psychosocial factors are associated with depressive symptoms in older adults with cancer. Our findings were consistent across two measures of depression (HADS-D and CES-D), strengthening the reliability and clinical relevance of the results. The study is further strengthened by its large sample size and the focus on adults aged 70 and older, a group often underrepresented in clinical trials and psychotherapy research. Finally, the clinical relevance of the findings represents a key strength. To our knowledge, this is the first study to examine multiple psychosocial pathways linking loneliness to depressive symptoms in OACs to highlight targets for clinical intervention. By identifying anxiety and demoralization as particularly robust correlates of depression, the study supports the use of multicomponent psychotherapeutic interventions to address existential distress, emotional regulation, and maladaptive aging beliefs, providing an important contribution to the geriatric psycho-oncology literature.

## Data Availability

The raw data supporting the conclusions of this article will be made available by the authors, without undue reservation.
